# The return of chloroquine-susceptible *Plasmodium falciparum* malaria in Zambia

**DOI:** 10.1186/s12936-016-1637-3

**Published:** 2016-12-05

**Authors:** Sydney Mwanza, Sudhaunshu Joshi, Michael Nambozi, Justin Chileshe, Phidelis Malunga, Jean-Bertin Bukasa Kabuya, Sebastian Hachizovu, Christine Manyando, Modest Mulenga, Miriam Laufer

**Affiliations:** 1 Tropical Diseases Research Centre (TDRC), 6th and 7th Floors, Ndola Central Hospital Building, P.O. Box 71769, Ndola, Zambia; 2 Division of Malaria Research, Institute for Global Health, University of Maryland School of Medicine, 685 West Baltimore Street, Baltimore, MD 21201-1509 USA

**Keywords:** Malaria, *Plasmodium falciparum*, Chloroquine, Anti-malarial resistance, PfCRT, Pyrosequencing, Zambia

## Abstract

**Background:**

*Plasmodium falciparum* resistance to anti-malarial drugs remains a major obstacle to malaria control and elimination. The parasite has developed resistance to every anti-malarial drug introduced for wide-scale treatment. However, the spread of resistance may be reversible. Malawi was the first country to discontinue chloroquine use due to widespread resistance. Within a decade of the removal of drug pressure, the molecular marker of chloroquine-resistant malaria had disappeared and the drug was shown to have excellent clinical efficacy. Many countries have observed decreases in the prevalence of chloroquine resistance with the discontinuation of chloroquine use. In Zambia, chloroquine was used as first-line treatment for uncomplicated malaria until treatment failures led the Ministry of Health to replace it with artemether-lumefantrine in 2003. Specimens from a recent study were analysed to evaluate prevalence of chloroquine-resistant malaria in Nchelenge district a decade after chloroquine use was discontinued.

**Methods:**

Parasite DNA was extracted from dried blood spots collected by finger-prick in pregnant women who were enrolling in a clinical trial. The specimens underwent pyrosequencing to determine the genotype of the *P. falciparum* chloroquine resistance transporter, the gene that is associated with CQ resistance.

**Results:**

Three-hundred and two specimens were successfully analysed. No chloroquine-resistant genotypes were detected.

**Conclusion:**

The study found the disappearance of chloroquine-resistant malaria after the removal of chloroquine drug pressure. Chloroquine may have a role for malaria prevention or treatment in Zambia and throughout the region in the future.

## Background

The emergence of *Plasmodium falciparum* resistance to anti-malarial drugs has thwarted malaria control efforts and remains a major obstacle to malaria elimination throughout the world. Chloroquine was one of the first drugs to be used on a wide scale for the treatment of malaria. Chloroquine resistance emerged independently in different geographic regions [1]. Chloroquine resistance that first emerged in Southeast Asia in the 1950s eventually reached sub-Saharan Africa in the 1970s. The spread of chloroquine-resistant falciparum malaria in Africa was responsible for a sharp increase in malaria morbidity and mortality [2, 3]. Resistance to chloroquine is modulated by the *P. falciparum* chloroquine resistance transporter (PfCRT) gene. A series of single-nucleotide polymorphisms is associated with increased rates of clinical failure [4]. The replacement of lysine with threonine at position 76 (K76T) is necessary for the observation of in vitro chloroquine resistance [5, 6].

As a result of the spread of chloroquine resistance and rising rates of clinical treatment failure, many countries changed the first-line drug treatment from chloroquine to sulfadoxine-pyrimethamine, either alone or in combination with chloroquine or amodiaquine. Sulfadoxine-pyrimethamine resistance quickly spread and now all malaria-endemic countries have adopted artemisinin-based combination therapy for the treatment of malaria. There is now evidence of the emergence of resistance to artemisinin derivatives and possibly their partner drugs in Southeast Asia [7–10]. The international community is taking urgent and definitive action to prevent the spread of resistance to the artemisinins and their partners to Africa, where that outcome would be even more devastating [11].

Due to widespread parasitological resistance and evidence of high rates of treatment failure with chloroquine, in 1993 Malawi became the first country in Africa to discontinue chloroquine use and adopted sulfadoxine-pyrimethamine for the treatment of uncomplicated malaria [12]. The prevalence of molecular markers of chloroquine-resistant malaria began to decrease immediately after its use was discontinued and by the turn of the century, there was almost no chloroquine-resistant malaria detectable throughout the country [13, 14]. A recent review demonstrated that decreases in the prevalence of chloroquine-resistant malaria were associated with decreases in chloroquine use as measured by demographic health surveys [15]. Since then, many countries have reported the return of chloroquine-susceptible malaria, but none has demonstrated a complete disappearance of chloroquine resistance as has been observed in Malawi [16–19].

In 2003, Zambia was the first country in sub-Saharan Africa to officially adopt artemisinin-based combination therapy for the treatment of uncomplicated malaria, discontinuing the use of chloroquine plus sulfadoxine-pyrimethamine and introducing artemether-lumefantrine [20]. Although the transition was challenged by limitations in the drug supply at the time, the country now has one of the longest history of artemisinin-based combination therapy use in the region. This study was designed to test the hypothesis that the prevalence of chloroquine resistance in Zambia would decrease or reach undetectable levels due to the long period of chloroquine discontinuation in the country.

## Methods

### Study design and participants

This cross-sectional survey utilized specimens collected from women enrolled in a randomized clinical trial from 2010 to 2013, evaluating the efficacy of different artemisinin-based combination therapy to treat malaria in women in their second and third trimester of pregnancy. The study design and results have previously been reported [21]. The criteria for enrolment in the study were: age ≥15 years old; gestation ≥16 weeks; *P. falciparum* mono-infection of any density, with or without symptoms; haemoglobin concentration ≥7 g/dL; residence within the health facility catchment area; willing to deliver at the health facility; and, ability to give informed consent. The study was carried out in Nchelenge district, Luapula province in the northern part of the country. This is an area of stable transmission with the entomological inoculation rate estimated at 4–48 infectious bites per 6 months in 2013 [22]. We collected and analysed specimens from pregnant women with a positive malaria smear who were enrolling the trial and before they received any antimalarial treatment. Comparisons were made to historical studies conducted in the Copperbelt and Central Province of Zambia. Although these areas are not geographically close to the study site, they are rural areas of Zambia and the results were expected to be similar to Nchelenge.

The participants were diagnosed with malaria by a rapid diagnostic test [(RDT) SD Bioline, Standard Diagnostics] that detects histidine-rich protein-II antigen. Drops of blood were collected and dried on 3 M Whatman filter paper. The specimens were collected from enrolled participants prior to any study treatment. Specimens with adequate quantities of blood were selected for molecular analysis.

### Molecular analysis

Parasite DNA was extracted from the dried blood spots. The specimens underwent nested PCR followed by pyrosequencing to genotype of position 76 in *Pfcrt*. Details of the protocol used for molecular analysis of the samples can be found on the website [23]. A summary of DNA extraction, amplification and sequencing is described here.

The DNA was extracted from each dried blood spot sample using a commercially procured kit (QIAamp^®^ DNA 96 Blood Kit, Qiagen), following a modified procedure for DNA extraction. The DNA was eluted in 150 μl of AE Buffer (Qiagen) and stored at −80 °C until time of use. Amplification of *Pfcrt* 76 was carried out using nested PCR. The primers are listed in Table 1. The PCR and pyrosequencing primers for the *Pfcrt* 76 gene were synthesized by IDT (Coralville, IA, USA). Primer sequences were designed using the Pyrosequencing™ Assay Design Software (Qiagen). PCR was performed using the Biorad T100™ and C1000 Touch™ thermocyclers (Bio-Rad, Hercules, CA, USA). The reaction volume for both the primary and nested PCR was 25 μL, and contained 1X PCR buffer (diluted from 10X Buffer, Qiagen), 200 μM mixture of dNTPs (Invitrogen), 2 mM MgCl2, (Qiagen), 1.5 units of HotStarTaq^®^ DNA Polymerase (Qiagen), 0.2 μM (0.8 μM for nested reactions) external forward and reverse primers and 1 μL of DNA. Thermal cycling conditions for the primary and nested PCR reactions were 95 °C for 15 min (HotStarTaq DNA Polymerase activation), followed by 40 cycles (25 cycles for nested reaction) with denaturation at 95 °C for 30 s, annealing at 45 °C for 45 s, and extension at 72 °C for 1 min; one cycle at 72 °C for 10 min and a final hold at 4 °C. Successful amplification was confirmed by running the nested PCR product and visualized on commercially procured E-gels (Invitrogen).

**Table 1:** Primers for amplification of the region of PfCRT 76

Codon	Primer	Sequence (5′–3′)	Bases	Amplicon size
72–97	External forward	GACCTTAACAGATGGCTCAC	20	347 bp
	External reverse	TTTTATATTGGTAGGTGGAATAG	23	
	Internal forward	Biotin-GGTAAATGTGCTCATGTGTTTAAACTTATT	30	241 bp
	Internal reverse	TTACTTTTGAATTTCCCTTTTTATTTCCA	29	
72–76	Pyrosequencing primer	AGTTCTTTTAGCAAAAATT	19	

For pyrosequencing, single-stranded biotinylated PCR products were prepared using the PyroMarkTM Vacuum Prep Tool and Workstation (Qiagen). 3 μL of Streptavidin Sepharose HP beads (Amersham Biosciences, Uppsala, Sweden) was added to 40 μL binding buffer (10 mM Tris–HCl, pH 7.6, 2 M NaCl, 1 mM EDTA, 0.1% Tween 20) and mixed with 2–5 μL PCR product (depending on the band intensity seen for the nested PCR product on the Qiaxcel) and 28 μL water for 5 min at room temperature using a thermomixer (Eppendorf) at a speed of 1400 rpm. The beads containing the immobilized template were captured on the filter probes of the Vacuum Prep Tool after the vacuum was applied and then washed with 70% ethanol for 15 s, denaturation solution (0.2 M NaOH) for 15 s, and washing buffer (10 mM Tris–acetate, pH 7.6) for 15 s. The vacuum was then released, and the beads were released into a PyroMarkTM Q96 HS Plate (Qiagen) containing 12 μL annealing buffer (20 mM Tris–acetate, 2 mM MgAc2, pH 7.6) and 0.4 μM sequencing primer. The plate was incubated at 80 °C for 2 min on a digital heat block, and allowed to cool at room temperature for 5 min. Pyrosequencing reactions were performed according to the manufacturer’s instructions using the PyroMark^®^ Gold Q96 Reagent Kit (Qiagen), which contained the enzyme, substrate and nucleotides. The assays were performed on the PyroMarkTM Q 96MD instrument (Qiagen). The sequence to analyse (STA) entered into the instrument was: G/TTA/TTT/CA/CATTACACA/TTACACTTAAATA. The nucleotide dispensation order used was: CGTATCATAGCACATGAC. The sample genotype was determined using the SNP mode of the PyroMarkTM Q 96MD software.

### Ethics, consent and permission

The study protocol was reviewed and approved by the Tropical Diseases Research Centre Institutional Review Board in Ndola, Zambia, and authority to conduct the research was sought in line with the existing Zambian national guidelines. Written informed consent was obtained from all individuals who agreed to participate in the study. A Material Transfer Agreement was signed prior to transferring anonymized specimens to the University of Maryland School of Medicine, Institute for Global Health, Division of Malaria Research laboratory.

## Results

Among the specimens from 900 participants who were recruited in the study, 314 dried blood spot filter papers had sufficient blood spot to be selected. DNA was successfully extracted, amplified and pyrosequenced for *Pfcrt* 76T in 302 samples. Clinical data were not available to the laboratory investigators.

**Fig. 1:**
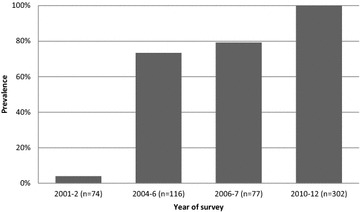
Prevalence of chloroquine-susceptible malaria in Zambia (PfCRT K76) in Zambia from 2001–2012 [23, 24]

All the 302 samples (302/302; 100%) analysed for the *Pfcrt* 76 harboured the wild type, susceptible AAA nucleotides, coding for lysine. No threonine was detected at position 76. The amino acid sequence in all specimens at positions 72-76 was CVMNK, the wild type, chloroquine-susceptible haplotype. Results from a decade of molecular surveys of PfCRT prevalence in Zambia are shown in Fig. 1.

## Discussion

Nchelenge District in Zambia is now only the second site to document the complete return of chloroquine-susceptible malaria after the removal of chloroquine drug pressure. This follows a slow decline in the prevalence of chloroquine resistance in Zambia: from 95% in 2001 [24] to 26% in 2006 [25] and is now at undetectable levels. Although historical molecular data are not available specifically from Nchelenge, clinical efficacy was reported in 1986–88. At that time, chloroquine failed to clear over 20% of infections in the first week, suggesting the molecular resistance marker estimates from neighbouring regions were accurate as molecular resistance rates are usually much higher than clinical failure rates [26].

To date, other countries have only demonstrated decreases in prevalence of chloroquine-resistant malaria. This may be due to several factors. In many countries, the use of chloroquine persisted for a longer period of time before artemisinin-based combination therapy was introduced, and also where the acquisition of anti-malarial drugs from the private sector is common. In addition, the return of chloroquine-susceptible infection likely occurs most quickly in the context of high transmission settings, where a large proportion of infections are not treated and there is ample opportunity for recombination during sexual reproduction in the mosquito [27]. The return of chloroquine-susceptible malaria in Malawi occurred via an expansion of susceptible parasites that had survived in the population despite chloroquine drug pressure [28]. In contrast, in Southeast Asia, where transmission is low, chloroquine-resistant malaria has become fixed in the population. As a result, even with changes in malaria treatment policy, chloroquine resistance continues to remain fixed in the parasite population.

There are several limitations to the generalizability of this study. Due to the sample size and the limited geographic region covered by the samples, this survey may have failed to detect rare cases of chloroquine-resistant malaria and the findings may be limited to the northern region where the study was conducted. The use of pyrosequencing is highly sensitive to detect minor genotype populations. Pyrosequencing can detect the presence of a second genotype that is present in 20% of the sample. Thus, the very low prevalence of chloroquine-resistant parasites cannot be entirely excluded. Finally, the samples were collected from pregnant women with infections detectable by RDT. Pregnant women are often used as sentinel groups for monitoring parasite prevalence and drug resistance due to their reliable contact with health facilities. Moreover, the drug-resistance patterns of their infections are unlikely to differ from the general population.

The return of chloroquine-susceptible malaria to the entire region may present a novel opportunity for re-introducing the use of chloroquine to prevent malaria, especially in vulnerable populations. It is a safe, well-tolerated and long-acting drug that can be administered in young children and also at any stage of pregnancy. Importantly, it does not have cross-resistance with current artemisinin-based combination therapy so the use of chloroquine would not compromise the efficacy of first-line treatment. With the spread of sulfadoxine-pyrimethamine resistance throughout eastern and southern Africa, chloroquine may be a viable alternative for intermittent preventive treatment or continuous chemoprophylaxis during pregnancy. Chloroquine may also be a reasonable option for use as seasonal malaria chemoprophylaxis in infants and children in regions where the combination of amodiaquine and sulfadoxine-pyrimethamine is not effective. Although Zambia, Malawi and Tanzania are areas where seasonal malaria chemoprophylaxis is likely to be effective [29], the program has largely been rolled out in West Africa, where sulfadoxine-pyrimethamine remains effective. The use of artemisinin-based combination therapy as intermittent treatment or chemoprevention could rapidly shorten the useful therapeutic life of the current first-line treatment in Africa. The confirmation of the resurgence of chloroquine-susceptible malaria may provide a new opportunity to use alternative medications to protect the most vulnerable populations.

## Conclusions

This study documented the disappearance of chloroquine-resistant malaria in northern Zambia after the adoption of artemisinin-based combination therapy. This is only the second region in the world where this phenomenon has been reported. These findings have important public health implications. Although chloroquine resistance may re-emerge if used as monotherapy for treatment of symptomatic disease, chloroquine may be a desirable option for prevention of malaria during pregnancy or for chemoprophylaxis in infants and children.

## Abbreviations

DNA: deoxyribonucleic acid
PCR: polymerase chain reaction
PfCRT: *Plasmodium falciparum* chloroquine resistance transporter gene
RDT: rapid diagnostic test
